# Accurate and Fast Numerical Estimation of Pattern Uncertainty for Mechanical Alignment Errors in High-Accuracy Spherical Near-Field Antenna Measurements

**DOI:** 10.3390/s25134227

**Published:** 2025-07-07

**Authors:** Kyriakos Kaslis, Samel Arslanagic, Olav Breinbjerg

**Affiliations:** 1 Department of Space Research and Technology, DTU Space, Technical University of Denmark, 2800 Kongens Lyngby, Denmark; saar@dtu.dk; 2 ElMaReCo, DK-1055 Copenhagen, Denmark; olavbreinbjerg@outlook.com

**Keywords:** antenna measurements, measurement errors, measurement uncertainty, uncertainty estimation

## Abstract

Every experimental measurement is affected by random and/or systematic error sources, causing the measurand to have an associated uncertainty quantified in terms of a confidence interval and confidence level. For high-accuracy spherical near-field antenna measurements, there are approximately 20 error sources whose individual contributions to the measurand uncertainty must be estimated for each antenna under test; thus, this uncertainty estimation is a required task in each measurement project. The error sources associated with the mechanical alignment of the antenna under test are of particular importance, not only because the consequential pattern uncertainty differs significantly for different antennas under test, but also because the common practice of experimental uncertainty estimation is very time-consuming with separate uncertainty measurements, thus requiring the antenna under test as well as the measurement facility. We propose a numerical pattern uncertainty estimation for mechanical alignment errors based on a nominal full-sphere measurement without the need for separate uncertainty measurements. Thus, it does not occupy either the antenna under test or the measurement facilities. In addition, numerical uncertainty estimation enables the isolation of individual error sources and their contributions to pattern uncertainties.

## 1. Introduction

Spherical near-field (SNF) antenna measurements imply that the near-field of an Antenna Under Test (AUT) is sampled by a calibrated probe over the surface of a surrounding sphere to evaluate the far-field radiation properties of the AUT [1]. Essential to the SNF is a mechanical positioning system, henceforth referred to simply as a positioner, which places the AUT and probe in relative positions as required by the spherical near-field transmission formula. There are many types of positioners [2]; in this work, we focus on the roll-over-azimuth positioner, which is common and is also present at the DTU-ESA Spherical Near-Field Antenna Test Facility [3].

High-accuracy SNFs are, like all physical measurements, affected by random and/or systematic error sources. For each AUT, and thus as part of each measurement project, it is necessary to estimate the contributions of these error sources to the measurand(s) of the AUT. There are approximately 20 sources of error in the SNF [4,5]. We focus here on errors originating from the mechanical alignment of the positioner since the associated uncertainties differ significantly for different AUTs and because of the significant impact of their uncertainty estimation on the measurement project.

The common experimental uncertainty estimation for these error sources consists of deliberately increasing the error beyond its typical value for it to become the dominant error, performing a full-sphere measurement with this increased error (a so-called uncertainty measurement), performing a near-field to far-field transformation, observing the change to the measurand, and linearly extrapolating the change down to the typical value of the error [1] (p. 270), [6]. Although straightforward, this experimental uncertainty estimation is very time-consuming, especially for electrically large antennas, as it requires the further availability of the AUT as well as the measurement facility, and it does not allow for full isolation of the individual error sources. The estimation of experimental uncertainty is often a time-limiting factor in high-accuracy SNF measurements.

The study of error analysis and uncertainty estimation in near-field antenna measurements is almost as old as the field itself, with early works naturally concerning the planar case [7,8] and leading to the now widely adopted NBS/NIST 18-term uncertainty budget [9]. For spherical near-field measurements, early works [6,10] led to a comprehensive account of error analysis in Hansen’s seminal monograph [1] (p. 216). Most of the later works regarding SNF have dealt with individual error sources or particular aspects of uncertainty estimation, but some have been of more general scope [4,11,12,13,14,15,16,17,18,19,20], and overviews are provided in [2,21]. From the outset [1,6,10] and in many of the aforementioned later works, numerical computations were used for error analysis and uncertainty estimation. However, these were typically general studies based on a computational model of a given typical AUT or for the purpose of a particular measurement range design; they were not meant to establish a procedure for AUT-specific and/or facility-specific numerical uncertainty estimation as an integral task for each measurement project.

In this work, we present and validate a numerical uncertainty estimation that overcomes the disadvantages of experimental uncertainty estimation by removing the requirement for additional full-sphere measurements. This applies to the three significant error sources of the mechanical roll-over-azimuth positioner, namely:

(A) Horizontal depointing at θ=0 (in short, θ-zero error),
(B) Axes intersection error (AIE),
(C) Probe transverse error (PTE).

Typical values for these errors, obtained from our experience measuring numerous antennas over the years [22,23,24,25,26] and their associated uncertainties from various projects at the DTU-ESA Facility, are listed in Table 1. These values (thousandths and hundredths of a dB) are indeed the typical uncertainty values of relevance for high-accuracy near-field antenna measurements, and they may well add up to tenths of a dB when all error sources are taken into account.

The numerical uncertainty estimation for the individual error sources is based on a mathematical model specific to the roll-over-azimuth positioner that determines the actual sampling points for the probe in the presence of the error. The probe signals at the actual sampling points are calculated using the spherical wave expansion for the AUT field obtained from the nominal full-sphere measurement and the spherical transmission formula. These actual probe signals, assigned to the nominal sampling points, are then used in the near-field-to-far-field transformation to observe the contribution of the error source to the uncertainty of the radiation pattern. For the validation of the models, we compare far-field pattern parameters, as opposed to the near-field probe signals, because it is typically the pattern parameters that are of practical interest.

Other sources of mechanical error include the orthogonality of the azimuth and roll axes, verticality of the azimuth axis, and depointing of the probe axis. However, from [1] (pp. 218–222) and our experience [22,23,24,25,26] at the DTU-ESA Facility, it has been assessed that these other errors can be controlled tightly enough, resulting in an insignificant influence on the measurands. It is noted that for many/most of the remaining error sources in SNF, such as the measurement distance, probe polarization ratio, and wave expansion truncation, the numerical uncertainty estimation is well established and widely employed.

Preliminary results of the numerical estimation method for spherical near-field antenna measurements were reported in [27,28]. However, new and significant developments that require further discussion are included in this manuscript, namely:

- The important distinction between θ-scans and ϕ-scans is addressed,
- Analytic formulas for the position of the probe in the case of a probe transverse error are given,
- More than 35 full-sphere computer simulations were conducted, and more than 15 full-sphere physical measurements were performed for validation, all producing very good results,
- Three very different (in terms of geometry and radiation pattern) antennas were used for the testing and validation of the developed theory,
- The experimental data from the tested antennas indicate that our proposed methods do not depend on the AUT, as predicted by theory.

The remainder of this manuscript is organized as follows. Section 2 defines the necessary terms and describes how uncertainties are usually estimated in practice, highlighting the disadvantages that this work mitigates. Section 3 introduces the theory behind the numerical estimation of θ-zero, axis intersection, and probe transverse errors. Section 4 presents the numerical and experimental results to validate the numerical uncertainty estimation and demonstrate its efficacy. Finally, the conclusions are presented in Section 5.

## 2. Experimental Uncertainty Estimation

In principle, any measurand, D, can be regarded as a function $D(r_{1},\ldots ,r_{N})$ of multiple random variables, $r_{i}$ (random variables are in bold). Since it depends on random variables, the measurand itself is a random variable. In regard to SNF, the measurand D could be the on-axis directivity, while the random variables $r_{i}$ would be the several parameters of the measurement system that influence the on-axis directivity, and for which individual uncertainties are assigned. Performing a measurement is tantamount to randomly selecting an instance of D from its range of possible values, and repeating the measurement could return a different value. This fact is ultimately the reason for the uncertainty regarding the true value of the measurand, i.e., the value if the variables were not random.

A common way to quantify the uncertainty in measurements is through the *p* confidence interval of the measurement, where *p* is the probability of finding the measurand in that interval ([29], pp. 307–308). This is also a method for assessing both the precision and accuracy of the measurement. In practice, there is a simple way to determine *p*. Assume that the Probability Density Function (PDF) of each random variable $r_{i}$, on which the measurand D depends, has been assessed. In practice, it is a sensible choice to assume a uniform PDF over a reasonable interval. For example, if an instrument that measures angles has a scale divided into integer degrees, then we can assume that a measurement of angles using this instrument produces a uniform random variable with a mean equal to the measurement result and a variance equal to 1/12 deg^2^.

The next step is to assume a linear relationship between the errors and the measurand. Although it is common practice to assume a uniform distribution of the error over a chosen interval as well as a linear dependence on this, such assumptions are not necessarily justified and could warrant separate investigations. Hence, the Taylor expansion of the measurand becomes

(1) $$D\left(r_{1},\ldots ,r_{N}\right)=D_{0}+\sum_{i=1}^{N}\frac{\partial D}{\partial r_{i}}r_{i}$$

where $D_{0}$ is the true value of the measurand, and the deterministic terms $\frac{\partial D}{\partial r_{i}}$ are the sensitivity coefficients [30].

The final step is to invoke the central limit theorem (CLT) ([29], pp. 278–279). The sum on the right side of (1) is a random variable that, according to the CLT, is normally distributed, with its mean being the sum of its individual means and its variance being the sum of their individual variances.

The PDF of the $r_{i}$ is known, so we now need to measure its sensitivity coefficient. This is done in practice by deliberately increasing the magnitude of an error to some value, say χ, in order to turn it into the dominating error of the measurement and, subsequently, performing an additional measurement. The new measurand is then compared with the measurand of a nominal measurement, where every effort has been made to suppress all error sources, and the difference divided by χ is the error’s sensitivity coefficient.

The advantages and disadvantages of this experimental uncertainty estimation method are clear. The major advantage is its simplicity and relatively straightforward implementation in virtually every measurement process. However, it is plagued by significant disadvantages.

- Complete measurements are necessary for every error source in order to evaluate the sensitivity coefficients,
- The AUT and the measurement facilities are occupied for an additional time,
- The error sources are not fully isolated from each other, thus diminishing the reliability of the method.

The primary disadvantage of the experimental estimation of uncertainty is the necessity to perform complete measurements to assess the sensitivity coefficient of each error source. Thus, alternative routes for uncertainty estimation that do not necessarily rely on additional measurements must be examined.

## 3. Numerical Uncertainty Estimation Results

The next three Section 3.1, Section 3.2 and Section 3.3 describe in detail how numerical uncertainty estimations can be performed for the three mechanical alignment error sources identified in the Introduction. Specifically, the actual sampling points caused by each of these three errors, which are different from the nominal sampling points when no error is present, are derived.

The central idea in the numerical uncertainty estimation method is to calculate the received probe signals in the case of a specific alignment error at the actual sampling points and, upon near-field to far-field transformation using these probe signals, calculate the error sensitivity coefficient for the far-field measurand without performing a separate uncertainty measurement. The probe signals are calculated with the spherical transmission formula [1]

(2) $$w\left(A,\chi ,\theta ,\phi \right)=\upsilon \sum_{\begin{array}{c} smn\\ \sigma \mu \nu \end{array}}T_{smn}e^{jm\phi }d_{\mu m}^{n}(\theta )e^{j\mu \chi }C_{\sigma \mu \nu }^{sn}(kA)R_{\sigma \mu \nu }$$

where w is the probe’s output signal, υ is the input signal of the AUT, $T_{smn}$ are the AUT’s transmission coefficients, $e^{jm\phi }$ are the rotation coefficients for the azimuthal angle ϕ, $d_{\mu m}^{n}$ are the rotation coefficients for the polar angle θ (so-called generalized Legendre functions), $e^{j\mu \chi }$ are the rotation coefficients for the probe orientation angle χ, $C_{\sigma \mu \nu }^{sn}(kA)$ are the translation coefficients for the probe to AUT distance A, k is the wavenumber, and $R_{\sigma \mu \nu }$ are the probe receiving coefficients; the ranges of the summation indices can be found in [1]. The rotation and translation coefficients are known mathematical functions, the probe receiving coefficients are known from probe calibration, and the measurement distance and AUT transmission coefficients are known from the nominal antenna measurement. In essence, (2) represents the translations and rotations required to make the AUT coordinate system coincide with the probe coordinate system.

**Figure 1:**
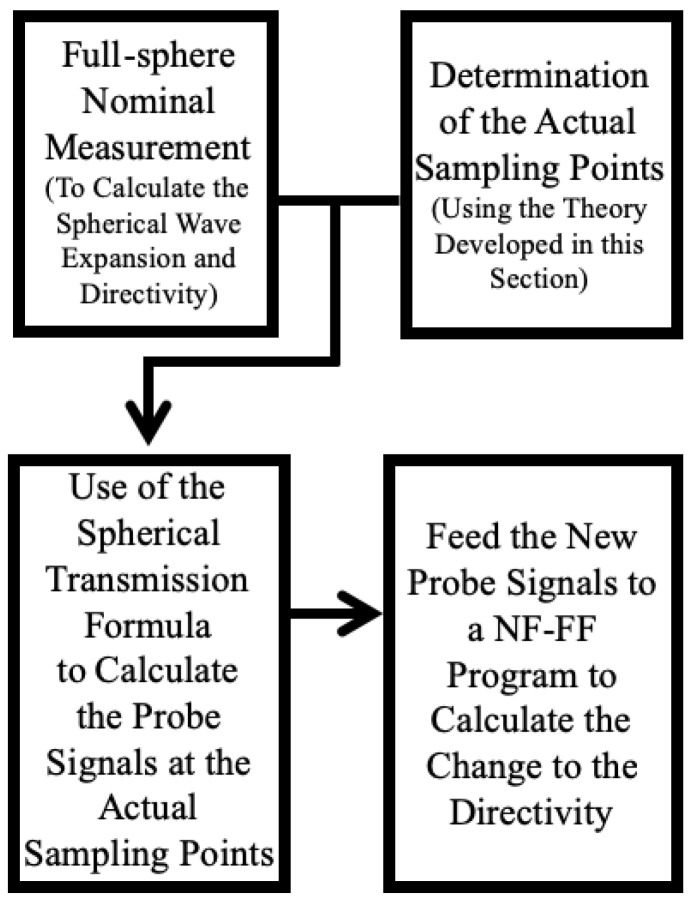
Flow chart showing the 4-step algorithm for numerical uncertainty estimation.

After the probe signals with the introduced alignment error are calculated, they are inserted into a spherical near-field to far-field transformation post-processing program (for example, SNIFT [31]) in order to evaluate the far-field radiation pattern and observe the difference in the measurand caused by this error. In this work, the measurand of interest is the on-axis directivity of the AUT. The entire procedure is summarized in the flowchart shown in Figure 1.

There are two traditional and widely used scanning schemes in SNF, namely θ-scans and ϕ-scans (see [1] (p. 190)). A θ-scan denotes a scheme where the θ (azimuth) angle rotates while the ϕ (roll) angle is stationary. When a full circle in θ has been performed, the ϕ angle steps and another full circle in θ is performed. In a ϕ-scan the scanning and stepping roles are reversed. As it will be shown later, the effect of the mechanical errors on the measurand is different for θ- and ϕ-scans.

### 3.1. Numerical Estimation of the θ-Zero Error

**Figure 2:**
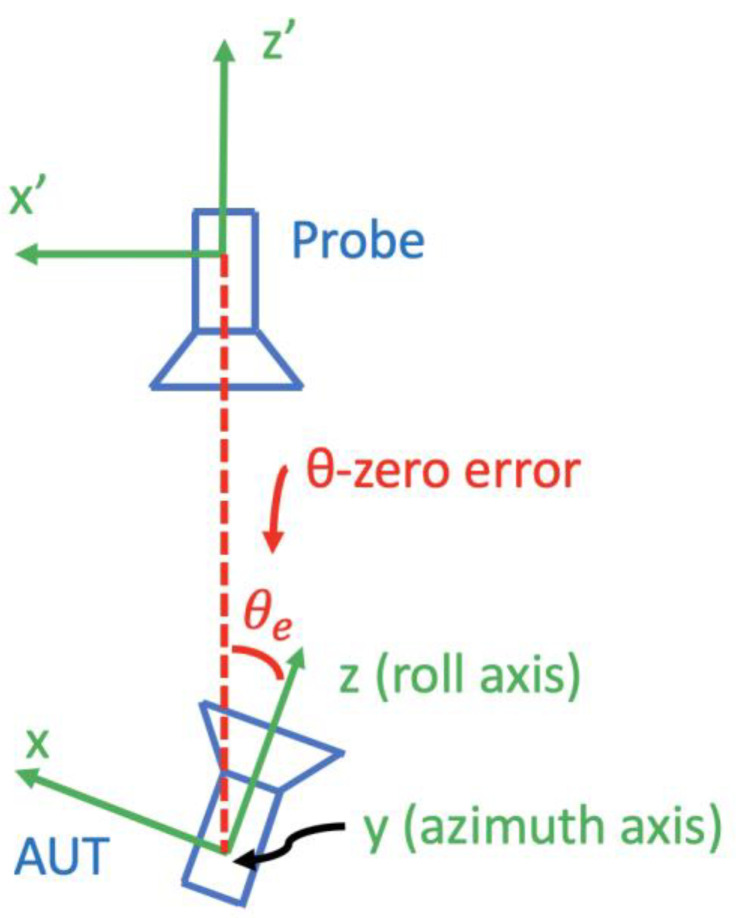
θ-zero error in a roll-over azimuth positioner (in a plane perpendicular to the azimuth axis). The roll axis is rotated with respect to the probe *z*-axis by an error angle $\theta_{e}$ when the azimuthal angle θ is defined to be zero.

Figure 2 shows the antenna measurement configuration of the roll-over-azimuth positioner in the plane perpendicular to the azimuth axis. Assume that no other error is present; then, if the θ coordinate is set to zero but the roll axis does not coincide with the *z’*-axis of the probe coordinate system, we say there is a θ-zero error.

**Figure 3:**
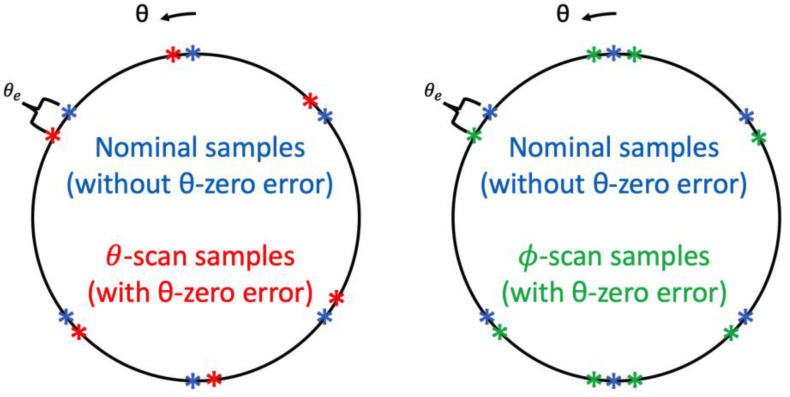
With a θ-zero error the samples are measured at actual sampling points different from the nominal positions, depending on the scanning scheme being a *θ*-scan or a *ϕ*-scan. In a *θ*-scan, the erroneous samples are ahead (in *θ*) of the nominal samples when *ϕ* < 180°, and lag behind when *ϕ* ≥ 180°. In a *ϕ*-scan, the erroneous samples are always ahead (in *θ*).

The θ-zero error affects the acquired samples differently, depending on the scanning scheme used. In the following, we discuss the different implications of this error for θ-scanning and ϕ-scanning, respectively. This point is easier to demonstrate using Figure 3. It shows a great circle through the poles of the measurement sphere with six representative sample points. The nominal samples, that is, the sample positions without a θ-zero error, are shown in blue. Assume there is a θ-zero error of magnitude $\theta_{e}$. If we use θ-scans, then the samples acquired would be the red ones, while if we use ϕ-scans we would get the green ones. In other words, in the case of a ϕ-scan, the actual azimuthal angle due to pointing error, $\theta_{pe}$, is simply the nominal angle θ plus $\theta_{e}$ (there is also a spread around the poles, which explains the additional points visible on Figure 3). However, in the case of a θ-scan, the actual azimuthal angle is

(3) $$\theta_{pe}=\left\{\begin{array}{c} \theta +\theta_{e},\;if\;0\leq \phi <\pi \\ \theta -\theta_{e},\;if\;\pi \leq \phi <2\pi \end{array}\right..$$

Since the probe signal w is a 2π-periodic function along any great circle, it has a Fourier series representation, namely:

(4) $$w(u)=\sum_{n=1}^{N}c_{n}e^{jnu}$$

where u is either θ or ϕ, and where the number of the modes, N, depends on the electrical radius of the AUT minimum sphere. The next step is to find the set of samples in case of a θ-zero error, $\theta_{e}$, which is equivalent to adding this error in (4). This is most efficiently performed by employing the Fast Fourier Transform (FFT) [32] in order to calculate the Fourier coefficients, $c_{n}$, and then compute (4) for any value of u. Since the computational complexity of FFT is O(NlogN) and the procedure needs to be performed for every ϕ coordinate, the total computational cost is $O(N^{4}{\left(logN\right)}^{2})$. It is to be noted that if we are only interested in the variations of the probe received signal with respect to θ or ϕ, then the spherical transmission formula can be written in the form of (4).

The last step is to feed the calculated probe signals in the presence of the θ-zero error to an SNF post-processing program, e.g., SNIFT, and find the difference, ∆D, of the directivity in comparison to the directivity obtained in the nominal measurement. The sensitivity coefficient of this error is then simply $\frac{∆D}{\theta_{e}}$.

### 3.2. Numerical Estimation of the Axes Intersection Error

From Figure 2, it is seen that there are two axes around which the AUT can rotate, namely the roll (ϕ-) axis and the azimuth (θ-) axis. If these two axes do not intersect, then we say that there exists an axis intersection error.

**Figure 4:**
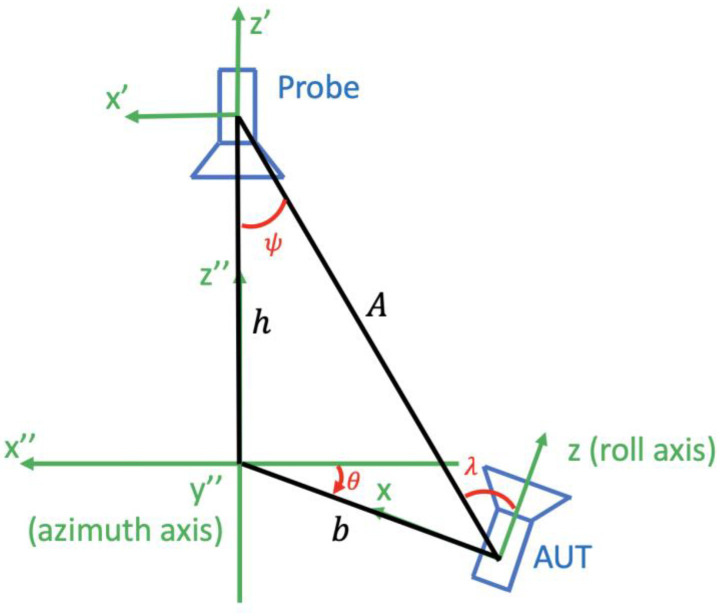
Axes intersection error in a roll-over azimuth positioner.

Figure 4 shows an antenna measurement configuration of a roll-over-azimuth positioner where an axes intersection error of magnitude b is present. In this case, the polar rotation angle is not θ but λ, the translation distance is not h but A, and an additional rotation around the angle ψ is needed; as detailed in [27], the transmission formula thus expands to take the form

(5) $$w\left(A,\chi ,\theta ,\phi \right)=\upsilon \sum_{\begin{array}{c} smn\\ \sigma \mu \nu \xi \end{array}}T_{smn}e^{jm\phi }d_{\mu m}^{n}\left(\lambda \right)C_{\sigma \mu \nu }^{sn}(kA)d_{\xi \mu }^{\nu }(-\psi )e^{j\xi \chi }R_{\sigma \xi \nu }.$$

For the additional rotation coefficient, the new index ξ is either 1 or −1, assuming the usage of a first-order probe. Similar to (2), (5) represents the series of rotations and translations needed to make the AUT coordinate system coincide with the probe coordinate system. Generally, it is a computationally intensive procedure to calculate the formulas of this type. However, making the reasonable assumption that the axes intersection error b is much smaller than the measurement distance h, (5) can be reduced to the computationally faster form of (2). One simplification was proposed in [27], and we present here an even more effective simplification. First, it can be proved through the law of cosines that for b≪A≈h

(6) $$A=\sqrt{h^{2}+b^{2}+2bhsin(\theta )}=h\left(1+O\left(\frac{b}{h}\right)\right)\approx h$$

and

(7) $$\psi =\arccos\left(\frac{h^{2}+A^{2}-b^{2}}{2Ah}\right)=\arccos\left(1+O\left({\left(\frac{b}{h}\right)}^{2}\right)\right)\approx 0.$$

For example, in the DTU-ESA Facility h is around 6 m and b is typically less than a tenth of a millimeter. Consequently, for small axes intersection error b, the signals received by the probe can be found using (2) by replacing θ with λ. Again, using the law of cosines, it can be shown that

(8) $$\lambda =\left\{\begin{array}{c} \arcsin\left(\frac{b+hsin\left(\theta \right)}{A}\right),\;0\leq \theta \leq \frac{\pi }{2}\\ \begin{array}{c} \pi -\arcsin\left(\frac{b+hsin\left(\theta \right)}{A}\right),\frac{\pi }{2}<\theta \leq \pi \\ \pi +\arcsin\left(\frac{b-hsin\left(\theta \right)}{A}\right),\pi <\theta \leq \frac{3\pi }{2}\\ 2\pi -\arcsin\left(\frac{b-hsin\left(\theta \right)}{A}\right),\frac{3\pi }{2}<\theta <2\pi \end{array} \end{array}\right..$$

The computational cost of numerically estimating the uncertainty of this error is $O(N^{5}logN)$. This is because one needs to perform an FFT to retrieve the Fourier coefficients of a nominally sampled scanning circle (cost O(NlogN)), use Equation (4) to calculate the samples for specific values of λ (this is because, according to (8), samples at non-equidistant locations need to be found, so FFT will not work and the cost is $O(N^{3})$) and, finally, this needs to be done for every scanning angle (cost O(N)).

An important point here that needs clarification is that, as with the θ-zero error, θ-scans and ϕ-scans will produce different sets of signals and, therefore, different predictions of the associated change in the measurand. That is because in θ-scans the azimuthal angle θ shown in Figure 4 takes values in the interval $\left[0,\;360\right)$ degrees, while in ϕ-scans it takes values in the interval [0, 180] degrees. The roll angle ϕ is irrelevant in the simulation of this error.

### 3.3. Numerical Estimation of the Probe Transverse Error

**Figure 5:**
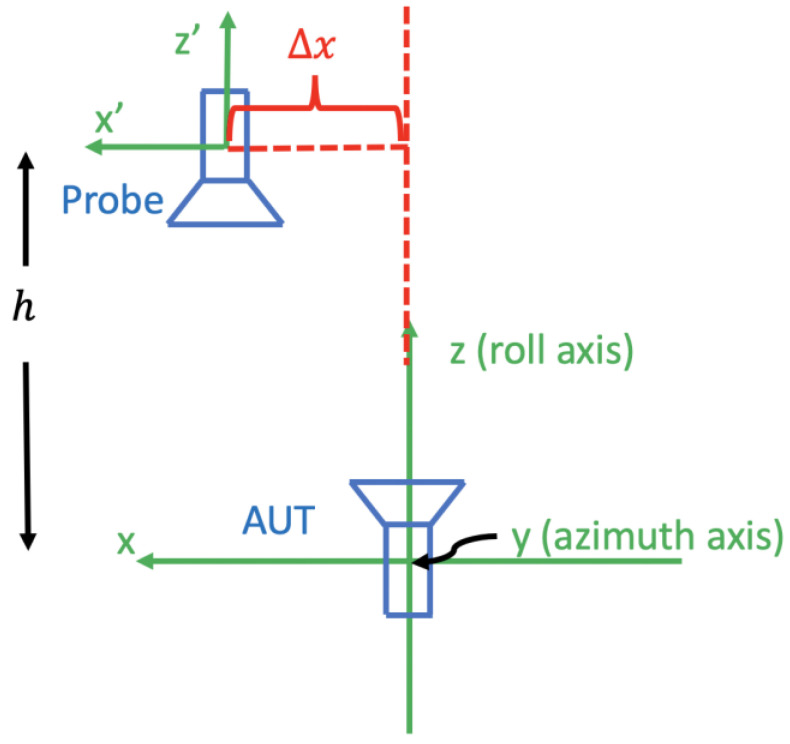
Probe transverse error in a roll-over azimuth positioner.

Assume that no other error is present; then, if the roll axis of the positioner is parallel to the *z’*-axis of the probe coordinate system but does not coincide with it when the azimuth axis is rotated to θ=0, then we say there is a probe transverse error. Figure 5 depicts an instance of a probe with a transverse error along the *x*-axis, ∆x. Note that it is a transverse displacement, as opposed to an angular displacement. Naturally, the error could be in any direction in the *x’y’*- plane of the probe coordinate system. For simplicity, we will analyze only the transverse errors along the *x*- and *y*-axes, as other transverse errors can be combined from these. Similarly to the θ-zero error, the two frequently used scanning schemes, namely the θ-scans and ϕ-scans, produce different actual positions when there is a probe transverse error along the *x*- or the *y*-axis.

For each of these cases, the exact formulas for the errors in the azimuthal and roll angles are provided in Appendix A, but they are not intuitive and easy to overview. With virtually no loss in accuracy, much simpler expressions can be derived in the case of small probe transverse errors, which is what is frequently encountered in practice, and are given in this section. To be more specific, in the case of a probe transverse error along the *x*-axis and a ϕ-scan, the first-order approximation of the error is

(9) $$\begin{array}{c} \theta_{pte}=\theta +\frac{∆x}{h}\\ \phi_{pte}=\phi \end{array}$$

where $\theta_{pte}$ and $\phi_{pte}$ are the actual azimuth and roll angles due to the probe transverse error, θ and ϕ are the nominal azimuth and roll angles, and h is the distance between the *x*-axis of the AUT coordinate system and the *x’*-axis of the probe coordinate system. In the case of a probe transverse error along the *y*-axis and a ϕ-scan, the first-order approximation of the error is

(10) $$\begin{array}{c} \theta_{pte}=\theta \\ \phi_{pte}=\phi +\frac{∆y}{hsin(\theta )} \end{array}.$$

Likewise, in the case of a probe transverse error along the *x*-axis and a θ-scan, it can be shown that the first-order approximation of the error is

(11) $$\begin{array}{c} \theta_{pte}=\left\{\begin{array}{c} \theta +\frac{∆x}{h},\;if\;0\leq \phi <\pi \\ \theta -\frac{∆x}{h},\;if\;\pi \leq \phi <2\pi \end{array}\right.\\ \phi_{pte}=\phi \end{array}$$

while in the case of a probe transverse error along the *y*-axis and a θ-scan, the first-order approximation of the error is

(12) $$\begin{array}{c} \theta_{pte}=\theta \\ \phi_{pte}=\left\{\begin{array}{c} \phi +\frac{∆y}{hsin(\theta )},\;if\;0\leq \phi <\pi \\ \phi -\frac{∆y}{hsin(\theta )},\;if\;\pi \leq \phi <2\pi \end{array}\right. \end{array}$$

It should be noted that these intuitive approximations are not all uniformly valid; i.e., (10) and (12) fail at the poles due to the sin(θ) in the denominator.

Since the effect of this error is to shift the location of the sampling points by a constant value, the computational cost is the same as for the pointing error, namely $O(N^{4}{\left(logN\right)}^{2})$.

## 4. Tests and Validation

Section 4 documents the numerical and experimental validation tests of the theory outlined in Section 3. Spherical near-field measurements are simulated using WIPL-D [33] based on the integral equation technique and are performed experimentally at the DTU-ESA Facility.

### 4.1. Simulated Validation Tests

**Figure 6:**
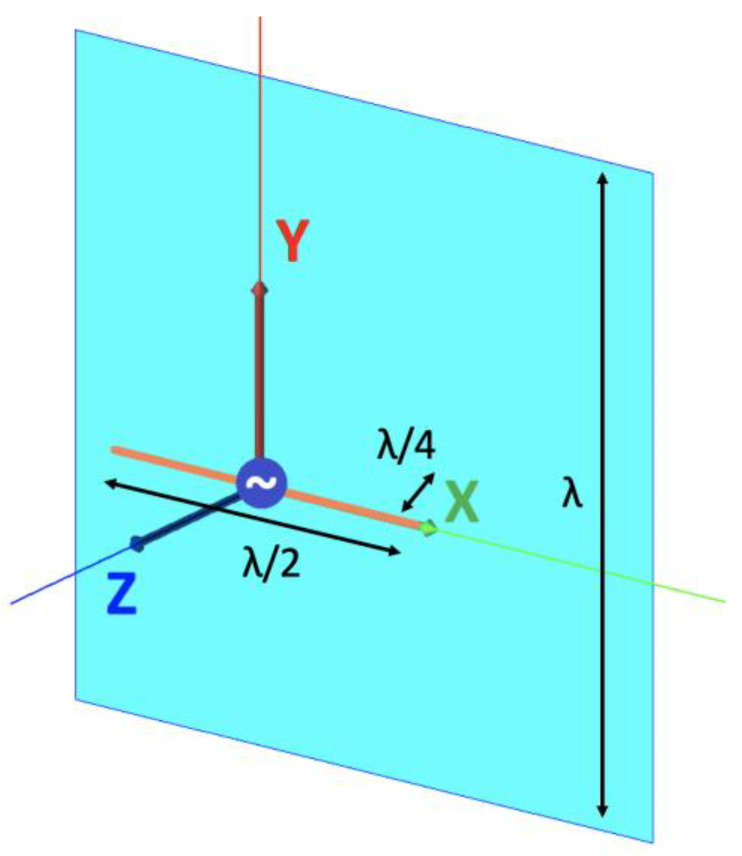
5 GHz (λ=6 cm) half-wavelength dipole with ground plane (GBD).

**Figure 7:**
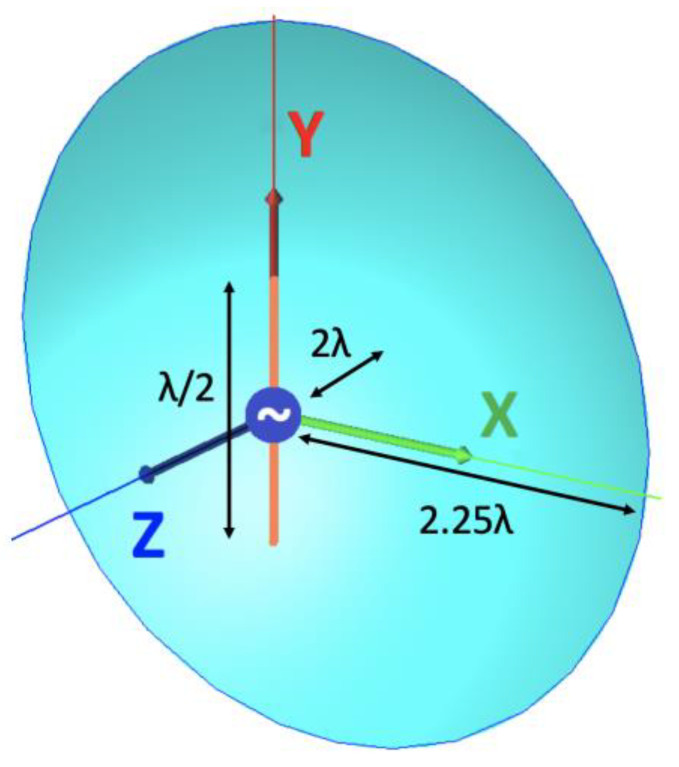
15 GHz (λ=2 cm) half-wavelength dipole with parabolic reflector (RA).

Two antennas were designed for the WIPL-D simulation testing. A 5 GHz half-wavelength dipole with a one-wavelength square ground plane at a distance of a quarter of a wavelength, called GBD, and a 15 GHz half-wavelength dipole with a parabolic reflector having a focal length of one wavelength, radius of two and a quarter wavelengths, and two wavelengths distance to the dipole, called RA, are used (see Figure 6 and Figure 7, respectively). The two antennas were designed to have distinct and different directivity patterns.

Two types of spherical near-field antenna measurements will be simulated in WIPL-D, both using ϕ-scans. For simplicity, the probe is chosen as a Hertzian dipole directly sampling the electric field at a measurement distance of 6 m. There are various advantages to this choice, such as avoiding multiple reflections, probe calibration, and other near-field effects, which are not relevant to this work. A distance of 6 m was selected because it is, roughly, the probe-AUT distance in the DTU-ESA Facility.

**Figure 8:**
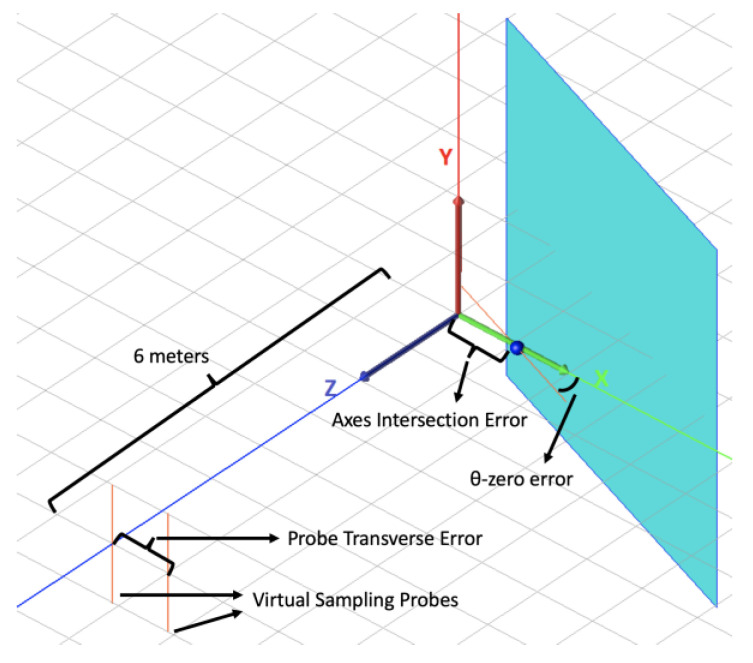
Alignment errors in SNF.

The first type of WIPL-D SNF simulation is called a *nominal measurement* because it is a full-sphere SNF without any alignment errors. The second type of WIPL-D SNF simulation is called *uncertainty measurement* of type PE/AIE/PTE (θ-zero/axes intersection/probe transverse error), depending on the error that has been implemented. This uncertainty measurement corresponds to the traditional experimental estimation of uncertainty. Only one error is introduced at a time, but for illustration purposes, Figure 8 shows GBD at the initial position $\left(\theta ,\phi \right)=(0,0)$ exhibiting all the aforementioned errors.

The theory in Section 3 is implemented in MATLAB (v. 2017b) code, which can accept the probe signals from the nominal measurement as input and produce the probe signals in the presence of an error of type PE/AIE/PTE of a specified magnitude as output. Then, the output is fed to SNIFT in order to compute the far-field measurand, which in all the following tests will be the AUT’s on-axis directivity. This constitutes a *numerical estimation*. The result of the numerical estimation is then compared to the result of the uncertainty measurement to validate the former.

**Figure 9:**
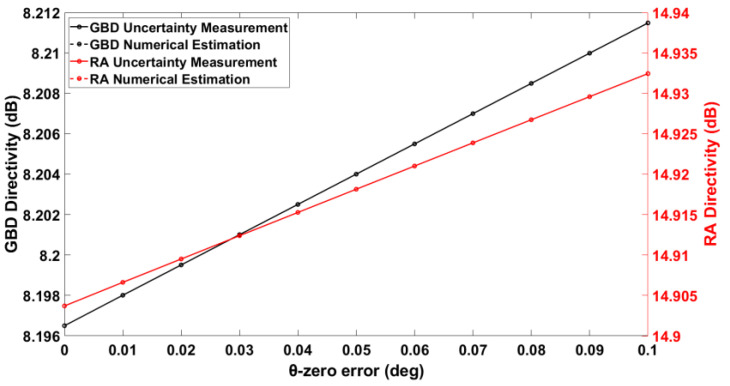
θ-zero error vs. on-axis directivity for both AUTs.

**Figure 10:**
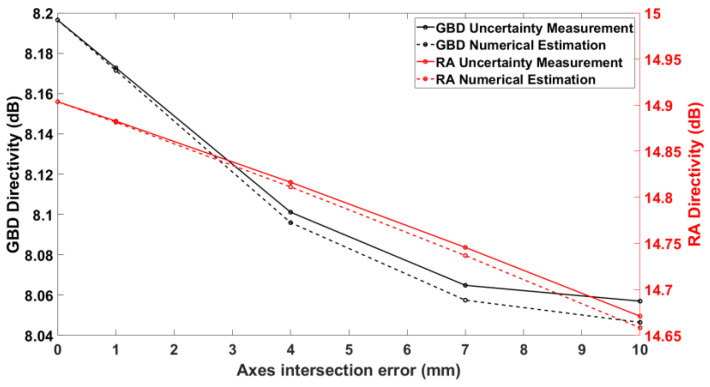
Axes intersection error vs. on-axis directivity for both AUTs.

**Figure 11:**
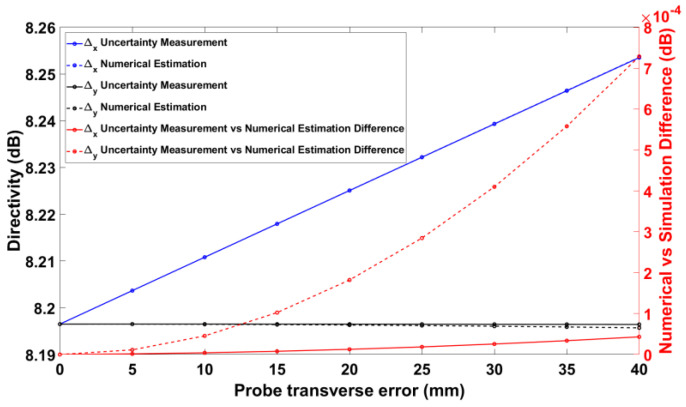
Probe transverse error vs. on-axis directivity for GBD.

**Figure 12:**
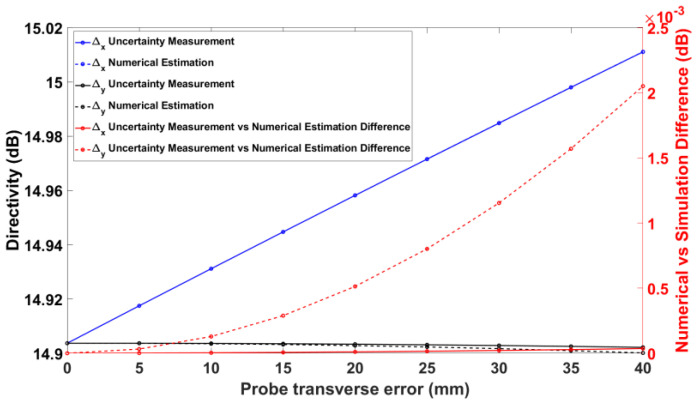
Probe transverse error vs. on-axis directivity for RA.

The results of the simulated validation tests are shown in Figure 9, Figure 10, Figure 11 and Figure 12. Figure 9 compares the directivities from the uncertainty measurement and the numerical estimation for each of the GBD and RA in the case of varying θ-zero error. It is found that the uncertainty measurement and the numerical estimation agree very well. This is justified by the fact that there are no approximations in the numerical estimation of this type of alignment error, as evident from (4). 

Figure 10 depicts a comparison between the uncertainty measurement and numerical estimation in the case of an axis intersection error. Here, the agreement is not as good as for the θ-zero case, but this was to be expected from the fact that approximations were employed, as shown in Equations (6) and (7). In any case, it is noted that the two types of simulation are still closer than 0.02 dB, even for axis intersection errors that are much larger than common practical values, confirming the validity of our theory.

Figure 11 shows the nominal and numerical on-axis directivities of the GBD when a probe transverse error is present. On the right vertical axis, the difference between these directivities is plotted in order to quantify the efficacy of the proposed methods. Similarly, Figure 12 shows the same data for the RA. It is noted that the numerical estimation is more accurate for the *x*-directed probe transverse error than for the *y*-directed probe transverse error. This can be explained by the fact that, as shown in Equations (9)–(12), the *y*-directed probe transverse error involves additional approximation steps due to its nature (it contains a singularity absent in the *x*-directed probe transverse error). As a result, the samples used for the numerical estimation are affected disproportionately since abrupt jumps in a periodic signal affect the samples everywhere. In any case, the differences in the directivity, as measured and predicted by numerical estimations, are less than 2 mdB, again documenting the validity of the numerical uncertainty estimation.

### 4.2. Experimental Validation Tests

**Figure 13:**
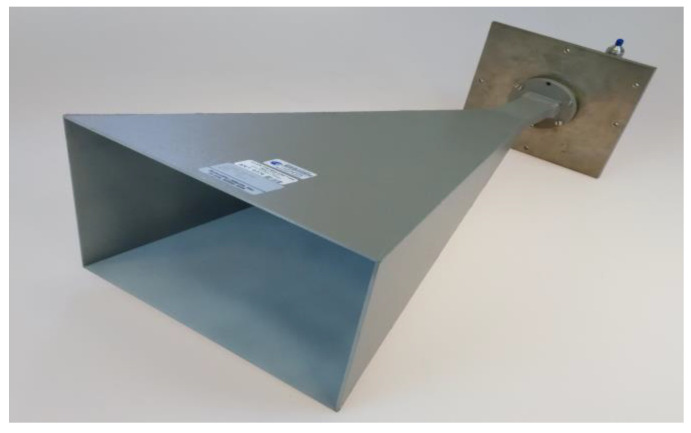
Scientific Atlanta 12-5.8.

The strategy for experimentally validating the developed theory is similar to the simulated validation described above. The standard gain horn, Scientific Atlanta 12-5.8 (depicted in Figure 13), operating at 8.35 GHz, was measured in the anechoic chamber of the DTU-ESA Facility, where, for the nominal measurement, every effort was made to reduce every error source as much as possible. Uncertainty measurements are then conducted, wherein θ-zero, axis intersection, and probe transverse errors of known magnitudes were deliberately introduced into the system to determine the extent to which these errors influence the measured on-axis directivity of the antenna. The probe used was a circular dual-port probe, called X1, which is calibrated for probe correction. The data was acquired with a θ-scan sampling every 4 degrees. Scanning at a speed of approximately 3 °/s, each full-sphere measurement required about 2 h to complete. The NSI-MI 9020 signal generator was used for transmitting, and the NSI-MI 750 receiver was used for receiving.

**Figure 14:**
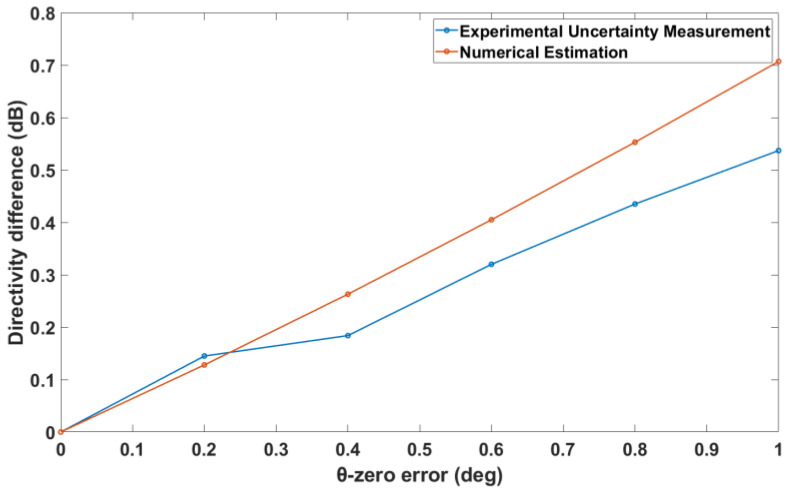
θ-zero error vs. on-axis directivity difference for standard gain horn.

**Figure 15:**
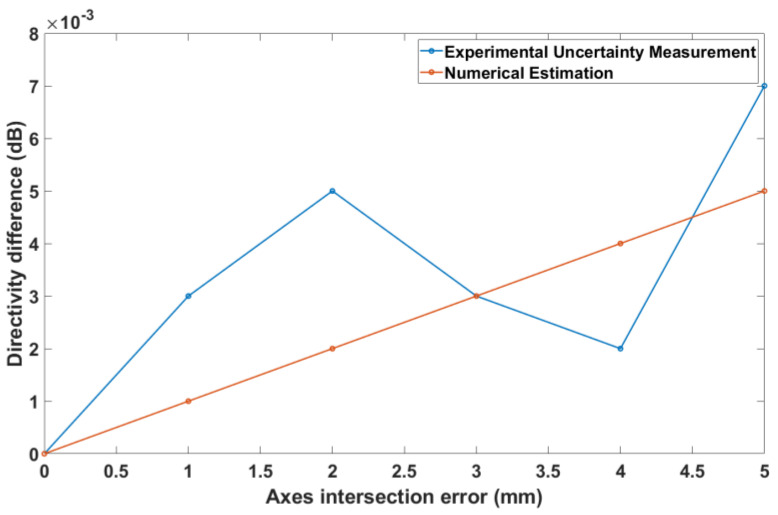
Axes intersection error vs. on-axis directivity difference for the standard gain horn.

**Figure 16:**
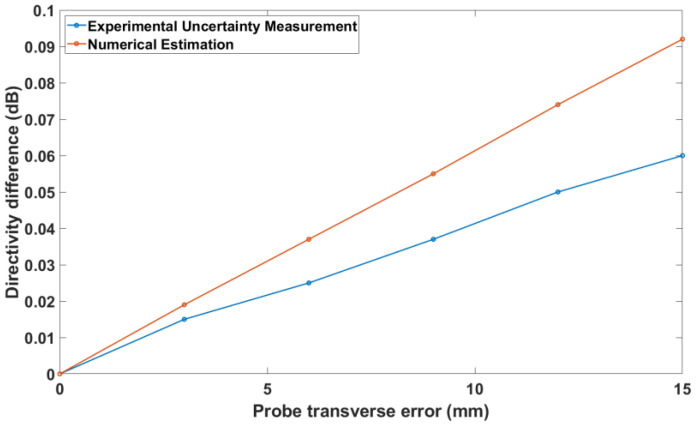
Probe transverse error vs. on-axis directivity difference for standard gain horn.

The experimental validation tests are shown in Figure 14, Figure 15 and Figure 16. These figures depict the absolute difference between the on-axis directivity of the AUT when measured without any known alignment errors (nominal measurement) and the on-axis directivity of the AUT when a certain alignment error was deliberately introduced. The directivity difference was determined from both the experimental uncertainty measurement and the numerical estimation theory described in Section 3.

Figure 14 shows the change in the directivity with increasing θ-zero error. The numerical estimation agrees with the experimental estimation of the rate of change of the on-axis directivity as a function of the θ-zero error, even though the selected errors are exaggerated in order to test the validity of the theory. 

The situation is somewhat different for the axis intersection error, as shown in Figure 15. Both estimations show a general tendency of increasing directivity difference, but the experimental method clearly fluctuates, indicating the presence of an additional error source. However, the difference between the numerical and experimental estimations is less than 0.003 dB, and it should be noted that the experimental uncertainty measurements always contain other sources of errors. Therefore, at these low levels, the small differences can be within the repeatability range.

Finally, Figure 16 depicts the directivity difference in the case of a probe transverse error. Again, it should be noticed that the agreement between numerical and experimental estimations is satisfactory, with a difference of less than 0.005 dB when the error is 3 mm, which can easily be attained in practice. The numerical estimations bound the change from above, providing a guaranteed maximum error estimation.

It is worth noting that if the agreement of the measurand (here, on-axis directivity) due to the investigated error sources is calculated in the range of the typical values shown in Table 1, a very good agreement of the order of 0.001 dB would be found. However, the antennas involved in both the simulations and physical experiments were simple in structure; therefore, they are only indicative of the efficacy of the proposed theory in a real-world scenario and do not provide direct proof. Nevertheless, it is quite encouraging that these results demonstrate that the proposed methods can be used in practice with satisfactory results.

## 5. Conclusions

In summary, for the mechanical alignment errors in SNF, numerical uncertainty estimation possesses several advantages over experimental uncertainty estimation: it is faster, separates the individual errors and uncertainties, and does not require the availability of the AUT or measurement facility. The numerical uncertainty estimation is based on a mathematical model of the actual sampling points for each error and each scanning scheme, but reuses the experimental results from the nominal measurements without the need for separate uncertainty measurements.

The numerical and experimental validation tests presented here document an excellent agreement of the order of 0.001 dB between the numerical and experimental approaches for the typical practical values of the θ-zero error, the axes intersection error, and the probe transverse error listed in Table 1—and a good agreement of the order of 0.01 dB even for much larger, and in practice seldom, values of these errors.

We have demonstrated the agreement in terms of directivities and directivity differences; from these, the sensitivity coefficients and actual uncertainties are readily obtained. In addition, the uncertainty of other measurands of interest, such as sidelobe levels and front-to-back ratios, can be readily calculated by employing the same approach demonstrated here for the directivity.

## Figures and Tables

**Table 1:** Standard Deviation of the AUT’s Directivity (in dB) due to θ-zero, Axes Intersection, and Probe Transverse Errors (Typical Practical Values of the Errors Listed).

AUT	θ-Zero (0.02°)	AΙΕ (0.05 mm)	PTE (0.3 mm)
Reflector Antenna (12 GHz)	0.008	0.000	0.000
Array Antenna (5.3 GHz)	0.001	0.011	0.004
Dual Array Antenna (10 GHz)	0.002	0.005	0.002

## Data Availability

Data are contained within the article.

## Appendix A

As it was mentioned in Section 3.3, the intuitive, but approximate, expressions that give the position of the probe with respect to the AUT in the case of a probe transverse error along the *y*-axis have a singularity for θ=0. In this appendix, we derive the exact equations that can be used to rectify these singularities.

Figure 5 shows the AUT and probe coordinate systems. The goal here is to find the actual spherical coordinates for the origin of the probe’s coordinate system with respect to the AUT coordinate system for arbitrary values of θ and ϕ in the presence of an alignment error.

In order to achieve this, we invoke a few results about spherical coordinate systems and the state of the probe coordinate system when a probe transverse error is present. Firstly, we represent with $R_{y}(\theta )$ the operator that rotates a vector in $R^{3}$ around the *y*-axis by an angle θ and $R_{z}(\phi )$ the operator that rotates a vector in $R^{3}$ around the *z*-axis by an angle ϕ. The exact matrix form of these operators can be found in [34] (p. 475) and are included here for simplicity, namely

(A1) $$R_{y}(\theta )=\left[\begin{array}{ccc} cos(\theta ) & 0 & sin(\theta )\\ 0 & 1 & 0\\ -sin(\theta ) & 0 & cos(\theta ) \end{array}\right]$$

(A2) $$\;\;\;R_{Z}(\phi )=\left[\begin{array}{ccc} cos(\phi ) & -sin(\phi ) & 0\\ sin(\phi ) & cos(\phi ) & 0\\ 0 & 0 & 1 \end{array}\right]$$

Secondly, the initial position (for θ=ϕ=0) of the probe coordinate system affected by a probe transverse error is

(A3) $$\left[\begin{array}{c} r^{\prime }\\ \theta^{\prime }\\ \phi^{\prime } \end{array}\right]=\left[\begin{array}{c} \sqrt{h^{2}+{∆x}^{2}}\\ {tan}^{-1}(\frac{∆x}{h})\\ 0 \end{array}\right]=>\left[\begin{array}{c} x^{\prime }\\ y^{\prime }\\ z^{\prime } \end{array}\right]=\left[\begin{array}{c} r^{\prime }sin(\theta^{\prime })\\ 0\\ r^{\prime }cos(\theta^{\prime }) \end{array}\right]$$

if there is a displacement ∆x toward the *x’*-axis and

(A4) $$\left[\begin{array}{c} r^{\prime }\\ \theta^{\prime }\\ \phi^{\prime } \end{array}\right]=\left[\begin{array}{c} \sqrt{h^{2}+{∆y}^{2}}\\ {tan}^{-1}(\frac{∆y}{h})\\ \frac{\pi }{2} \end{array}\right]=>\left[\begin{array}{c} x^{\prime }\\ y^{\prime }\\ z^{\prime } \end{array}\right]=\left[\begin{array}{c} 0\\ r^{\prime }sin(\theta^{\prime })\\ r^{\prime }cos(\theta^{\prime }) \end{array}\right]$$

if there is a displacement ∆y toward the *y’*-axis. This is shown in Figure 5.

In order to find the Cartesian coordinates of the probe coordinate system with respect to the AUT coordinate system, we apply the rotator operators, $R_{y}(\theta )$ and $R_{z}(\phi )$ to (A3) and (A4). If applied properly, the order in which the operators are applied is immaterial. The values of the θ and ϕ depend on the scanning scheme used. Based on the description of the two different scanning schemes (see [1] (p. 190)), we deduce, after some calculations, that an *x*-directed probe transverse error has Cartesian coordinates

(A5) $$\left[\begin{array}{c} x^{\prime \prime }\\ y^{\prime \prime }\\ z^{\prime \prime } \end{array}\right]=r^{\prime }\left[\begin{array}{c} \sin\left(\theta^{\prime }\right)\cos\left(\phi \right)\cos\left(\theta \right)+\cos\left(\theta^{\prime }\right)\sin\left(\theta \right)cos(\phi )\\ \sin\left(\theta^{\prime }\right)\cos\left(\theta \right)\sin\left(\phi \right)+\cos\left(\theta^{\prime }\right)sin(\theta )\sin\left(\phi \right)\\ -\sin\left(\theta^{\prime }\right)\sin\left(\theta \right)+\cos\left(\theta^{\prime }\right)\cos\left(\theta \right) \end{array}\right]$$

where θ∈[0,π] and ϕ∈[0,2π) for ϕ-scans and θ∈[0,2π) and ϕ∈[0,π) for θ-scans.

Similarly, for the case of a *y*-directed probe transverse error the Cartesian coordinates become

(A6) $$\left[\begin{array}{c} x^{\prime \prime }\\ y^{\prime \prime }\\ z^{\prime \prime } \end{array}\right]=r^{\prime }\left[\begin{array}{c} \cos\left(\theta^{\prime }\right)\cos\left(\phi \right)\sin\left(\theta \right)-\sin\left(\theta^{\prime }\right)sin(\phi )\\ \cos\left(\theta^{\prime }\right)\sin\left(\theta \right)\sin\left(\phi \right)+\sin\left(\theta^{\prime }\right)\cos\left(\phi \right)\\ \cos\left(\theta^{\prime }\right)\cos\left(\theta \right) \end{array}\right]$$

where θ∈[0,π] and ϕ∈[0,2π) for ϕ-scans and θ∈[0,2π) and ϕ∈[0,π) for θ-scans.

We would like now to find the spherical coordinates of the origin of the probe coordinate system affected by a transverse error, call them $\left(r^{\prime \prime },\theta^{\prime \prime },\phi^{\prime \prime }\right)$. It is obvious that $r^{\prime \prime }=r^{\prime }$. For $\theta^{\prime \prime }$ and $\phi^{\prime \prime }$ we obtain (from [34] (p. 177))

(A7) $$\theta^{\prime \prime }=arccos\left(\frac{z^{\prime \prime }}{r^{\prime }}\right)$$

and

(A8) $$\phi^{\prime \prime }=arctan\left(\frac{y^{\prime \prime }}{x^{\prime \prime }}\right)$$

It should be noted that $arccos(x):\left[-1,\;1\right]\to [0,\;\pi ]$ and $arctan(x):\left[-\infty ,+\infty \right]\to [-\frac{\pi }{2},\frac{\pi }{2}]$. For the case of $\phi^{\prime \prime }$ this is problematic, because in the usual spherical coordinate system the range of $\phi^{\prime \prime }$ is [0, 2π). The results given below consider this detail and provide the proper spherical coordinates.

Expanding these equations using (A5) and (A6) we get that an *x*-directed probe transverse error has spherical coordinates

(A9) $$\theta^{\prime \prime }=\left\{\begin{array}{c} \arccos\left(\frac{hcos\left(\theta \right)-∆xsin\left(\theta \right)}{r^{\prime }}\right),\;0{}^{\circ}\leq \theta \leq \pi \\ \arccos\left(\frac{hcos\left(\theta \right)+∆xsin\left(\theta \right)}{r^{\prime }}\right),\;\pi <\theta <2\pi \end{array}\right.$$

(A10) ϕ″=ϕ+π, y″<0 ^ ϕ<πϕ−π,y″>0 ^ ϕ≥πϕ, otherwise

where the symbol ‘^’ designates the logical AND. Similarly, a *y*-directed probe transverse error has spherical coordinates

(A11) $$\theta^{\prime \prime }=arccos\left(\frac{hcos(\theta )}{r^{\prime }}\right)$$

(A12) ϕaux=arctanhsinθsinϕ+∆y cosϕhcosϕsinθ−∆y sin(ϕ) 

(A13) ϕ″=ϕaux, x>0 ^ y ≥0ϕaux+π, x<0ϕaux+2π, x>0 ^ y<0π2,x=0 ^ y>03π2,x=0 ^ y<0ϕ,x=y=0 

Note that, unlike their small-error counterparts, there are no singularities or ambiguities in Equations (A9)–(A13).
